# Supplementary material for: IQGAP2 Inhibits Migration and Invasion of Gastric Cancer Cells via Elevating SHIP2 Phosphatase Activity
Source: Int J Mol Sci. 2020 Mar 13;21(6):1968. doi: 10.3390/ijms21061968 (PMC7139352; doi:10.3390/ijms21061968)
Supplement: Supplementary file 1 [file ijms-21-01968-s001.pdf]

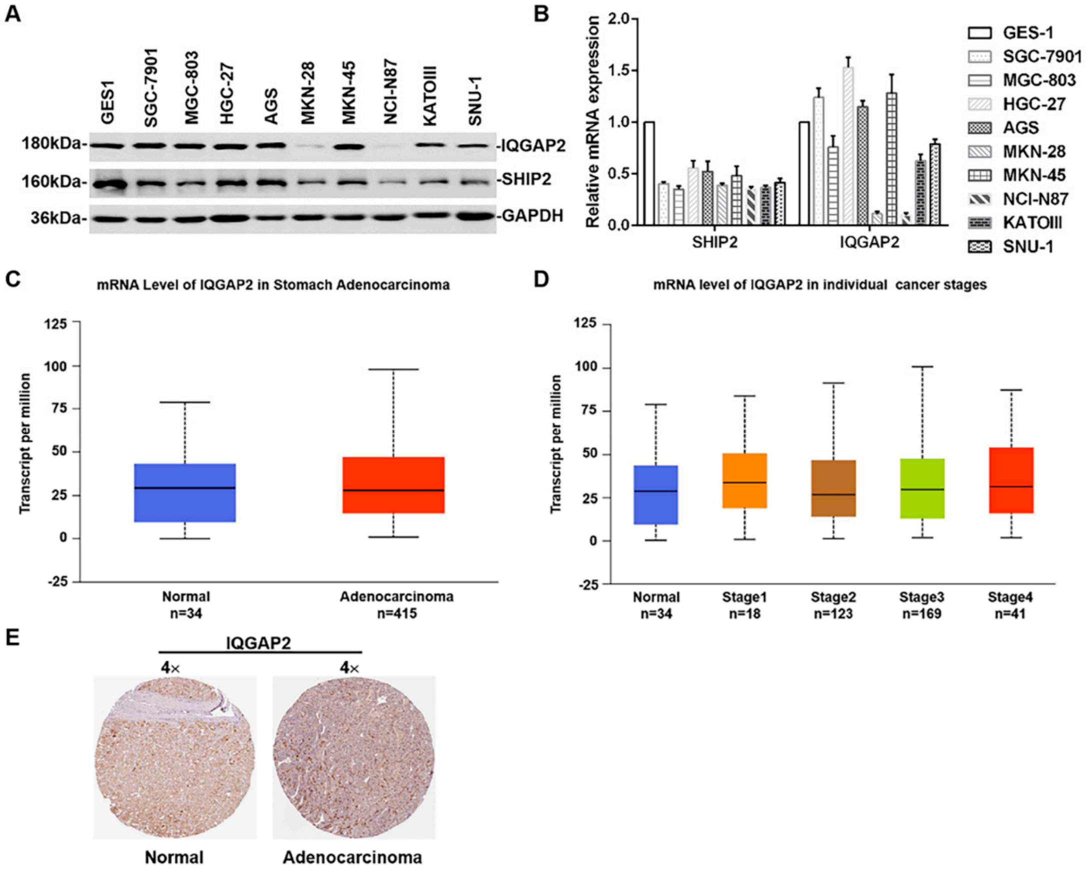

Figure S1. Expression of IQGAP2 and SHIP2 in human GC. (A) Whole cell lysates from the normal gastric mucosa epithelial cells GES-1and a panel of GC cells were subjected to Western blot analysis. Data shown are representative of three individual experiments; (**B**) The mRNA levels of SHIP2 and IQGAP2 from GES-1 cells and a panel of GC cells were detected by qRT-PCR. The relative abundance of target gene mRNA expression in GES-1 was arbitrarily designated as 1 (n = 3, mean ± SEM) ; (**C**) The mRNA levels of IQGAP2 in tissue samples of stomach adenocarcinoma were compared with those in normal tissues. Images were obtained from UALCAN database (http://ualcan.path.uab.edu) ; (**D**) The mRNA levels of IQGAP2 in tissue samples at different individual clinical stages were compared with those in normal tissues. Images were obtained from TCGA database using UALCAN platform (http://ualcan.path.uab.edu); (**E**) Representative images of immunohistochemical staining of IQGAP2 (brown) on stomach adenocarcinoma specimens and normal stomach tissues were obtained from HPA database (http://www.proteinatlas.org).
